# “Cancer changes everything, but it makes you wonder—am I still enough?” serial focus groups with adolescent and young adult cancer patients to understand experiences with cancer and sexual and reproductive health

**DOI:** 10.1186/s12885-025-14561-7

**Published:** 2025-08-01

**Authors:** Niki Oveisi, Vicki Cheng, Dani Taylor, Preet Kang, Lori A. Brotto, Stuart Peacock, Helen McTaggart-Cowan, Gillian E. Hanley, Sharlene Gill, Meera Rayar, Amirrtha Srikanthan, Mikaela Barnes, Haydn Bechthold, Norman Jansen, Mary A. De Vera

**Affiliations:** 1 Faculty of Pharmaceutical Sciences, University of British Columbia, 2405 Wesbrook Mall, Vancouver, BC V6T 1Z3 Canada; 2 Collaboration for Outcomes Research and Evaluation, Vancouver, BC Canada; 3 Patient Research Partner, Vancouver, BC Canada; 4 Faculty of Medicine, University of British Columbia, Vancouver, BC Canada; 5 BC Cancer, Vancouver, BC Canada; 6 Faculty of Health Sciences, Simon Fraser University, Burnaby, BC Canada; 7 Faculty of Medicine, University of Ottawa, Ottawa, ON Canada; 8 Department of Medicine, Division of Medical Oncology, The Ottawa Hospital, Ottawa, ON Canada; 9 The Ottawa Hospital Research Institute, Ottawa, ON Canada; 10 Centre for Health Evaluation and Outcome Sciences, Vancouver, BC Canada

**Keywords:** Sexual health, Reproductive health, Cancer survivorship, Adolescent and young adult, Cancer treatment

## Abstract

**Background:**

The impacts of cancer and its treatment on sexual and reproductive health among adolescents and young adults (AYA, ages 15–39) are not understood. We conducted a patient-oriented, novel serial focus group study to explore the sexual and reproductive health experiences of AYA cancer patients during and beyond treatment.

**Methods:**

Participants (≥ 18 years) who were diagnosed with cancer between 15 and 39 years and reside in Canada were assigned to respective focus group cohorts based on shared characteristics (e.g., gender, cancer stage). Each cohort participated in three serial focus groups that emulated support groups, fostering a sense of community. Focus groups were co-facilitated with a patient research partner (PRP) and guided by a topic guide co-created with PRPs. Framework analysis involving steps of familiarizing with transcripts, identifying thematic framework, indexing data sections by theme, extracting and organizing data into charts, and analyzing charts, were applied to transcripts.

**Results:**

Altogether, 48 AYA cancer patients, divided into 8 cohorts, participated in a total of 24 focus groups. Cohorts included: cis-gender women (*n* = 10), 2SLGBTQIA+ (*n* = 7), Black, Indigenous, and People of Colour (BIPOC; *n* = 7), cis-gender men (*n* = 6), breast cancer (*n* = 6), pelvic cancer (*n* = 6), diagnosed during adolescence (ages 15 to 19; *n* = 4), and stage 4 (*n* = 3) (one individual participated in two cohorts). Cohorts had representation of nonbinary and gender fluid participants (*n* = 4), non-heterosexual sexual orientation (*n* = 14), and racial diversity (*n* = 12). The final framework encompassed two thematic areas broadly categorized as patients’ experiences (“what is happening to them”) and their responses (“how they are reacting or acting”). The first thematic area (three themes), focused *inward* on the direct effects of cancer and its treatment on patients’ sexual and reproductive health. The second thematic area (three themes), looks *outward* at the external impacts, capturing how cancer affects aspects of patients’ sexual and reproductive health, including relationships, societal expectations, and community support.

**Conclusion:**

Uncovering the profound and complex personal and interpersonal impacts of AYA cancer on sexual and reproductive health, our study has important implications for informing appropriate and affirming support.

**Supplementary Information:**

The online version contains supplementary material available at 10.1186/s12885-025-14561-7.

## Introduction

Over the past 50 years, studies have shown a nearly 30% increase in cancer incidence among adolescents and young adults (AYAs), defined as individuals aged 15–39 [1]. Treatments are increasing rates of remission – however, the effects of both cancer and its treatment are often lifelong [2–4], leading to long-term challenges across psychosocial [5–8] and sexual and reproductive health domains [9, 10]. 

The World Health Organization defines sexual and reproductive health as “a state of physical, emotional, mental and social well-being in all matters relating to: sexuality and the reproductive system functions and processes [sexual health]/[reproductive health]” [11]. In prior systematic reviews and meta-analyses, our team quantified associations between AYA cancer and sexual (e.g., vaginal dryness and ejaculatory dysfunction) [12] and reproductive (e.g., use of fertility treatment and gestational diabetes) [13] health outcomes. These findings underscore the physical dimensions of cancer’s impact on sexual and reproductive health.

On the other hand, there are psycho-oncological considerations related to sexual and reproductive health. However, to date, qualitative research that provides contextual understanding of AYA cancer patients’ experiences with sexual and reproductive health and care is limited. Indeed, few qualitative research studies, mostly involving interviews with female AYA cancer patients, have identified challenges with reproductive and/or sexual health, such as infertility [14, 15], sexual dysfunction [16], and body dysmorphia due to visible impacts of cancer and its treatment [17]. Other critical limitations include the underrepresentation of males and a siloed approach that fails to recognize the interlinked nature of sexual and reproductive health. Finally, given the importance of peer support and shared experiences among AYA cancer patients [18, 19], qualitative methods that harness collective experiences within a group (e.g., focus groups) may uncover insights not possible through individual interviews. To address these gaps, we conducted serial focus groups with diverse participants to explore the sexual and reproductive health experiences of AYA cancer patients during and beyond treatment.

## Methods

### Study design

We conducted qualitative research using a constructivist paradigm of inquiry, acknowledging that knowledge is socially constructed and context-dependent [20]. Patient research partners (PRPs), who have lived experience with AYA cancer, either in remission or still undergoing treatment, contributed to the development of the topic guide, co-facilitated focus groups, and supported data analysis and interpretation (Additional file 1) [21]. This study was approved by the Behavioural Research Ethics Board at the University of British Columbia (H21-03591).

### Participants

We recruited participants online from 2023 to 2024. through social media campaigns, including the Twitter, Instagram, and Facebook, of research team members, affiliations, PRPs, and organizational partners. The recruitment posts were initially made once, but if we did not achieve sufficient reach for certain groups or identities, we reposted and reached out again to maximize visibility and engagement. We also used cancer registry mailing lists to reach out to potential participants once over the study. Overall, we made efforts to reach a broad range of AYA cancer-related groups, including those representing various cancer types, stages, and demographic characteristics, to ensure diverse participation in the study. Individuals were eligible if they were: (1) diagnosed with cancer between 15 and 39 years; (2) 18 years or older at time of consent; (3) residing in Canada; and (4) able to communicate in English. Individuals who expressed interest accessed the study website to complete the online consent form and questionnaire, using the survey platform Qualtrics [22]. This questionnaire consisted of questions on sociodemographic (e.g., gender, sexual orientation, current age, race/ethnicity, etc.) and clinical (e.g., cancer type, stage, age at diagnosis, treatment, etc.) characteristics. We used iterative purposive sampling to select participants representing various demographic and clinical factors including age, gender, cancer type, and stage of treatment.

### Data gathering

**Fig. 1:**
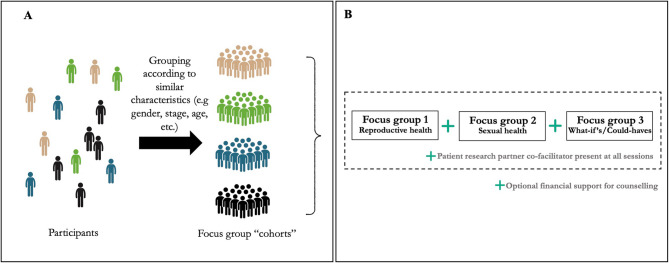
Study design including (**A**) Creation of focus group cohorts and (**B**) Serial focus groups

We created focus group cohorts based on participants’ shared characteristics gathered in the questionnaire (e.g., age, gender, cancer type, and stage of treatment). Cohort creation was an iterative process: as themes and distinctions emerged during focus groups, additional cohorts were formed to address specific experiences and needs. Importantly, participants did not switch between cohorts; rather, new participants were recruited to form additional cohorts. This flexible, participant-driven approach ensured that the study remained responsive to the insights and experiences of the participants. Each cohort participated in three serial focus groups (~ 2 h each), designed to foster a sense of trust and community over time, which enhanced the quality of the data collected (Fig. 1). While these groups were not designed to be therapeutic, the serial nature of the focus groups helped create an environment conducive to open discussion, These sessions were conducted via a licensed version of Zoom [23]. Focus groups were co-facilitated by a AYA cancer researcher (NO), and a PRP. Topics explored in the three groups were: 1) reproductive health (e.g., fertility preservation, family planning); 2) sexual health (e.g., sexual functioning, intimacy); and 3) reflections on care received for sexual and reproductive health concerns during cancer treatment and beyond. Data gathering and analysis took place concurrently, following an iterative process where the analysis guided the ongoing data collection, continuing until thematic saturation was reached, wherein no new themes, patterns, or insights were emerging from the data [24]. Honoraria were offered and, finally, recognizing the sensitivity of the topics discussed, we provided participants with financial support for counseling services should they choose to access them.

### Analysis

Audio recordings were transcribed verbatim by a third-party transcriptionist and imported into the qualitative data management software program, NVivo 12 [25]. We applied framework analysis, which involved the following steps: (1) establishing familiarity with the transcripts; (2) identifying a thematic framework by combining themes arising from the data through judgments about meaning, relevance, and the importance of issues; (3) indexing sections of the data corresponding to specific themes; (4) extracting data from its original textual context and placing it in charts with the headings and subheadings emerging from the thematic framework; and (5) analyzing the data in the charts to map and interpret concepts, isolate associations, and generate explanations for the findings [26]. Framework analysis was chosen for its combination of inductive and deductive approaches; this approach enabled us to incorporate both established and emerging themes, and the resulting framework is applicable for informing real change in healthcare and policy. Three researchers completed analysis (NO, VC, and PK), with a fourth assisting in resolving conflicts (MADV). We also applied sex and gender-based analyses to understand how sex, gender, and gender-related factors (e.g., roles, relations, identities) could influence individuals’ experiences with sexual and reproductive health throughout recruitment and analysis [27]. 

## Results

### Participant characteristics

Altogether, we recruited 48 AYA eligible cancer patient participants over ~ 18 months, mainly from social media recruitment. The participants were divided into 8 cohorts, participated in a total of 24 focus groups (Table 1). Cohorts included: cis-gender women (*n* = 10), 2SLGBTQIA+ (*n* = 7), Black, Indigenous, and People of Colour (BIPOC; *n* = 7), cis-gender men (*n* = 6), diagnosed with breast cancer (*n* = 6), diagnosed with pelvic cancer (*n* = 6), diagnosed during adolescence (ages 15–19; *n* = 4), and stage 4 cancer (*n* = 3).

With respect to cancer characteristics, at time of cancer diagnosis, the median age of participants was 31 years old; and at time of study participation, 33 years old. Participants reported having multiple primary sites, the most common of which were breast (*n* = 15), colon (*n* = 8), and brain (*n* = 3). At participation, 68.8% of participants had completed cancer treatment. With respect to sociodemographic characteristics, particularly race, most participants were White (75%), followed by East and South Asian (6.3% each), and West Asian (4.2%). There was a skew towards female sex (77.1%) and cis-gender women (70.8%), with representation from non-binary (6.3%) and gender fluid folks (2.1%). Sexual orientation was diverse, with heterosexual (64.6%) and bisexual (14.6%) highest, followed by asexual (8.3%), demisexual (4.2%), homosexual (4.2%), and pansexual (4.2%).

**Table 1:** Focus group cohorts and participant characteristics (*N* = 48)^A^

Focus group cohort	Number of participants in each		
Cohort 1: Cis-gender women	10 (20.8)		
Cohort 2: 2SLGBTQIA+	7 (14.6)		
Cohort 3: BIPOC	7 (14.6)		
Cohort 4: Cis-gender men	6 (12.5)		
Cohort 5: Diagnosed with breast cancer	6 (12.5)		
Cohort 6: Diagnosed with pelvic cancer	6 (12.5)		
Cohort 7: Diagnosed during adolescence (15–19 years)	4 (8.3)		
Cohort 8: Diagnosed with Stage 4 cancer	3 (6.3)		
Participant characteristics			
Sociodemographic characteristics		Cancer characteristics	
Age at cancer diagnosis, median (range)	31 (15–39)	Primary cancer location, *n*^B^	
Age at participation, median (range)	33 (21–48)	Breast	15
Race, *n* (%)		Colon	8
White	36 (75.0)	Brain	4
East Asian	3 (6.3)	Non-Hodgkin’s Lymphoma	3
South Asian	3 (6.3)	Cervical	3
West Asian	2 (4.2)	Thyroid	3
Indigenous x White	1 (2.1)	Ovarian	2
South Asian x West Asian	1 (2.1)	Hodgkin’s Lymphoma	2
South Asian x White	1 (2.1)	Leukemia	2
West Asian x White	1 (2.1)	Sarcoma	2
Sex assigned at birth, *n* (%)		Testicular	1
Female	38 (79.2)	Neuroendocrine	1
Male	10 (20.8)	Lung	1
Gender, n (%)		Spinal	1
Woman	34 (70.8)	Nasopharyngeal	1
Man	10 (20.8)	Rectal	1
Nonbinary	3 (6.3)	Uterine	1
Gender fluid	1 (2.1)	Stage, *n* (%)	
Sexual orientation, *n* (%)		1	8 (16.7)
Heterosexual	31 (64.6)	2	11 (22.9)
Bisexual	7 (14.6)	3	15 (31.3)
Asexual	4 (8.3)	4	9 (18.8)
Demisexual	2 (4.2)	Missing	5 (10.4)
Homosexual	2 (4.2)	Treatment status, *n* (%)	
Pansexual	2 (4.2)	Completed	33 (68.8)
Relationship status, *n* (%)		Currently undergoing	14 (29.2)
Married	17 (35.4)	Not yet started	1 (2.1)
Single	15 (31.3)		
Common-law or co-habiting	7 (14.6)		
In a relationship	6 (12.5)		
Dating	2 (4.2)		
Separated	1 (2.1)		
Education, *n* (%)			
Graduated from a 4-year program	22 (45.8)		
Post-graduate degree	15 (31.3)		
Graduated from a 2-year program	5 (10.4)		
Attended some college and/or university	5 (10.4)		
Secondary or highschool	1 (2.1)		
Religion, *n* (%)			
No religious affiliation	30 (62.5)		
Christian-protestant	6 (12.5)		
Christian-catholic	4 (8.3)		
Spiritual	4 (8.3)		
Muslim	3 (6.3)		
Jewish	1 (2.1)		
Income (CAD), *n* (%)			
<30,000	5 (10.4)		
30,001–60,000	7 (14.6)		
60,001–90,000	7 (14.6)		
90,001–120,000	15 (31.3)		
120,001–150,000	5 (10.4)		
150,001+	9 (18.8)		
Location, *n* (%)			
Ontario	18 (37.5)		
British Columbia	17 (35.4)		
Manitoba	7 (14.6)		
Newfoundland and Labrador	3 (6.3)		
Alberta	2 (4.2)		
Nova Scotia	1 (2.1)		
Employment, *n* (%)			
Employed full-time (40 + hours)	18 (37.5)		
On disability	12 (25.0)		
Student	5 (10.4)		
Unable to work	4 (8.3)		
Employed part-time (< 40 h)	3 (6.3)		
Self-employed	3 (6.3)		
Unemployed	3 (6.3)		

*Abbreviation*: *BIPOC* Black, Indigenous, People of Colour

^A^One participant was a member in two focus group cohorts

^B^Some participants had more than one primary site of cancer

### Framework analysis

**Fig. 2:**
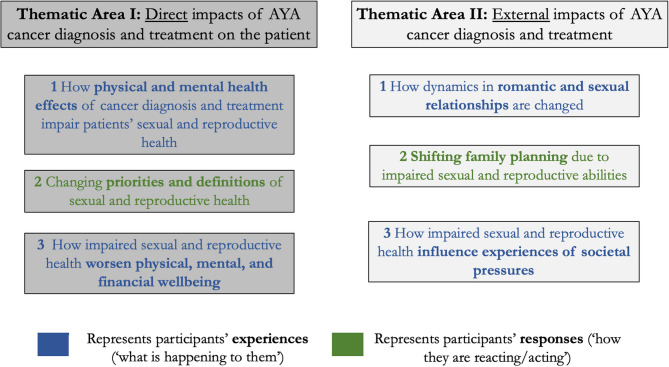
Final framework involving thematic areas and corresponding themes

The final framework included two thematic areas broadly described as participants’ experiences of (‘what is happening to them’) and responses to (‘how they are reacting/acting’) the impacts of cancer diagnosis and treatment on sexual and reproductive health (Fig. 2). The first thematic area, which comprises three themes, looks *inward* at direct impacts on sexual and reproductive health of patients themselves. Conversely, the second thematic area, which comprises three themes, looks *outward* and captures the external impacts of cancer diagnosis and treatment on aspects related to the sexual and reproductive health of patients, including their relationships and how they experience societal expectation. Below, we describe each theme with evidence from participants’ lived experiences as shared in the focus groups.

### Thematic area I: direct impacts of AYA cancer diagnosis and treatment on the patient

#### Theme 1: how the physical and mental health effects of the cancer diagnosis and treatment impaired patients’ sexual and reproductive health (experience)

Participants shared how physical changes due to cancer treatments distorted body image and self-esteem contributed to diminished sexual health. Participants described negative body image impacts caused by hair loss and weight fluctuations with one noting, “*I lost half my hair and cancer caused me to gain a lot of weight… you feel like an alien in your own body*”. Another participant shared the concern of visible scars and physical changes, “*I have some pretty bad scarring on my neck and hyperpigmentation*” which resulted in a loss of confidence and self-esteem in intimate settings, noting “*that’s all [the partner] is going to see*”. Some participants described physical changes from cancer treatments involving temporary or permanent colostomy bags that also diminished sexual health. One participant shared, “*when [they] have a piece of [their] intestine sticking out of [their] body and a bag of poop stuck to [their] abdomen*,* [they] don’t want people to touch [them] or see [them] naked*” (Cohort 1, colorectal cancer at 36 years old).

Participants also described how cancer treatments led to physical challenges that limited their ability to engage in sexual activity. Physical challenges experienced during cancer treatment included “*lockjaw*”, “*extreme fatigue*”, or having to “*keep [their] leg completely straight for a total of nine weeks*”. Aside from feeling ill-prepared for these challenges, participants shared how these were disabling with respect to sexual health as they “*had not even consider[ed] what it would be like trying to have sex as a very disabled person*”. Some physical challenges persisted beyond cancer treatment, leading to long lasting impairments to sexual health. An example shared was vaginal atrophy or stenosis among females, which a participant with colorectal cancer described as when the “*vagina basically closes up*,* and the depth and width completely changes.*” This participant did not receive timely treatment and was later informed that “*young people find it helpful to use dilators during chemotherapy*.” Unfortunately, this advice came after their treatment was completed, making the damage irreversible and permanently affecting their ability to engage in penetrative sex.

Participants described interconnected experiences with mental health and sexual health. Desire, libido, arousal, and enjoyment were significantly lower than usual due to hormonal changes, fatigue, and emotional distress. In terms of desire and libido, participants described “*having virtually no sex drive*”, and that “*during treatment [sex] just wasn’t a thing*”. One participant articulated how “*the whole cancer diagnosis and treatment brought feelings of loss of control of [their] body*” and that they “*felt very unsafe in [their] own body*”. Arousal and enjoyment were also impaired, with one participant with breast cancer describing that “*when [they] lost [their] whole breast and nipple*,* [they] can’t physically climax anymore*”. The impact of mental health on sexual health is well-captured by this quote from a participant that “*if your mental health is not there*,* you can’t really get into your sexual health*”. Conversely, participants also shared that the physical changes and challenges of impaired sexual health often led to feelings of depression or anxiousness. This interconnectedness of mental health and sexual health is described by a participant here: “*it is a bidirectional thing because I’ve always dealt with anxiety and depression*,* but it’s been a lot more acute since the cancer diagnosis. So*,* not being able to perform or be there*,* have the motivation sexually makes me doubt myself as well and I think is a manifestation of the depression I’ve been feeling but it also contributes to those feelings as well because it does make me feel like a lesser man even though I know intellectually that’s not the case. It’s just a very frustrating thing - it’s definitely impacted my mental health and how I view myself*” (Cohort 4, brain cancer at 34 years old).

Effects of cancer treatments on fertility and consequent challenges of fertility preservation profoundly impacted participants’ reproductive health, with varying experiences among females and males. Female participants, particularly those who underwent treatment to the pelvic or cranial area, shared experiences with induced early menopause and associated physical (i.e., hot flashes, night sweats, insomnia) and mental (i.e., anxiety, brain fog) symptoms. Many echoed the sentiment of early menopause as “*the worst side effect of [the] entire cancer experience*”. Female participants also shared experiences with fertility preservation as a long and emotionally taxing process which was not always successful leading to “*grieving the kids that [they] didn’t get to have twice*”. Some female participants also described the risks with fertility preservation, including delayed cancer treatment. Male participants described varying impacts to their sperm count and reproductive ability but found that sperm freezing and storage was a viable option. Across the sexes, participants shared that providers were unable to offer detailed information on fertility preservation outcomes due to lack of research on fertility in AYA cancer, which increased feelings of anxiety. Participants who underwent fertility preservation shared that despite uncertainties “*it’s [their] one shot to have [their] own kid*”. One participant shared challenges and difficulties of being pregnant during their cancer treatment: “*It messed up my mental health. I [was] always dreaming of a normal pregnancy and seeing myself going through the treatment and how much my body changed…you have a growing belly and you should be glowing*,* but I was so sick and bald and miserable and it wasn’t a happy time. It was hard because we were at the age when our friends would have babies and you would see [these] picture perfect tummies and they’re taking pictures and enjoying it and I just couldn’t enjoy anything. I’m forever grateful that I have a baby now and he’s healthy*,* but I won’t ever have a baby again”* (Cohort 2, breast cancer at 27 years old).

#### Theme 2: changing priorities and definitions of sexual and reproductive health (response)

Cancer diagnosis and treatment forced participants to abruptly change their sexual and reproductive priorities. Shifting priorities, such as foregoing having sex or the possibility of conceiving children, were common at the start of treatment. Some participants describing entering “*survival mode*” rather than maintaining sexual quality of life or preserving family planning. With respect to sexual priorities, a participant described that “*sex [was] not a priority*” and something that they *“would not bring up or be confident about anymore.”* Reproductive priorities also changed for participants as several shared that they “*always thought [they] had time*,* [they] could freeze [their] eggs if [they] wanted to later but then [they] got cancer*”. These shifts in priorities often came suddenly as many participants were forced to make major decisions regarding their sexual and reproductive health within shortened timelines, with little to no information to support these decisions. Participants shared that they “*didn’t have any conversation about [sexual and reproductive health] or the impact it would have on [their] body*”. This was particularly heightened in those diagnosed with stage 4 cancer, where sexual and reproductive health was deemed less urgent than competing concerns regarding disease prognosis. AYA cancer patients often felt unprepared for these shifting priorities.

With shifting sexual and reproductive priorities, participants were often forced to change how they viewed and defined sexual and reproductive health. Some described a broadened definition to include more diverse ways of having sex or growing their family, while others realized that their definition of what matters to them is much narrower than before cancer. These definitions for sexual and reproductive health were often closely linked to one’s sense of identity. A common example was changes in the ability to conceive and carry children as captured by a participant who described waking up after hysterectomy and feeling that “*it’s official… I’ve lost a piece of myself*”. While some considered mental health and social well-being as part of their definition of reproductive health, others thought it to be “*the ability to conceive a child or the ability to have functional sperm*”. The importance of choice and autonomy over their sexual and reproductive health was also highlighted, especially when cancer involves “*a loss of choice*”, and that “*everyone should have that choice*”. Although participants were “*forced to think about [sexual and reproductive health] in ways [they] never thought they would*”, they found that over time they became more comfortable in their shifting definitions and were able to self-advocate with their healthcare providers. Depending on how broad or limited a participant described their definition of sexual and reproductive health, this related to less or more plasticity in coping with changes.

Across genders, shifts in priorities and definitions of sexual and reproductive health were associated with an emotional and mental health toll. Cis-gender men participants described the mental aspect of “*having the confidence to still be able to perform*”, which fluctuated greatly during and after treatment. Cis-gender women participants described how their perception of gender had to shift when they “*felt like less of a woman…[as they] might not be able to give [their] partner the family they want*” and they “*didn’t feel like a woman*,* and [they] feel like [they] don’t have any value without that reproductive piece and the sexual piece and just everything that goes along with that*.” Participants, particularly those in treatment or recently completed treatment, used words such as “*betrayal*” to describe how their relationship with their body has changed since their cancer diagnosis. They sometimes viewed “*the cancer and body as if they were one and the same instead of viewing [their] body as an ally*”. However, participants beyond treatment and in remission described “*trying to make peace with [their] body by reminding [themselves] all the things it has done*” through cancer diagnosis and treatment. In this “*relearning*”, participants described “*rediscovering [their] body in the same way [they] did when [they] were going through puberty*” and being a “*born again virgin*”. The importance of past sexual and reproductive health experiences on current priorities and definitions was also highlighted, with reflections on “*the context [they] come in*,* the identities held*,* the previous sexual relationships*,* and how it all interplays*”. Many had made peace with new timelines and sexual and reproductive health capabilities, describing “*trying to put on [their] old life and it didn’t fit*”, and realizing that “*it’s not necessarily about going back*,* it’s about creating something new with these new experiences and new body*”.

#### Theme 3: how impaired sexual and reproductive health worsens physical, mental, and financial wellbeing (Experience)

Theme 1 captures sexual and reproductive health complications experienced by patients with AYA cancer that are largely due to physical changes, physical challenges, and mental health toll. Theme 3 depicts how these experiences with sexual and reproductive health with cancer, in turn, further affects physical (e.g. bone density loss and cardiovascular issues), mental (e.g. grief and trauma), and financial (e.g. costs of fertility preservation) wellbeing. The physical impacts of changes to sexual and reproductive health can stay with patients for years after treatment. For people assigned female at birth who experience early menopause, concerns include bone density loss and cardiovascular risks, especially given the limited research on the lifelong outcomes of AYA cancer females, and “*fertility isn’t just the ability to have kids*,* there is a lot more to it*”. Participants also described long-lasting grief and trauma relating to sexual and reproductive health complications and preservation, stating that “*even ten years later when it comes to my sexual health and my relationship to it*,* there’s a ton of grief there. There’s a lot of grief for all the years where*,* I couldn’t*,* I just couldn’t*” and that “*more than missing sex itself*,* I missed wanted to have sex…well*,* who am I now? Because I’m no longer defined by a lot of things I used to define myself as.*” A consistent example of trauma among female participants was the fertility preservation process, which was described as “*a very traumatic experience*” when they “*go inside of you*,* vaginally…talking about being inside of me while I’m just there*,* and I never wanted to take part in that*”. These experiences shaped sexual quality of life, so that “*whenever [they] thought about sex*,* [they] thought about that*”. Participants who also experienced previous sexual trauma described “*that the whole cancer diagnosis and treatment brought back the feeling of loss of control in the body and being in a physically compromised position”.* This interlink of sexual trauma and reproductive health underscores how deeply intertwined these aspects of health are; changes in one area can reverberate through the other, exacerbating both physical and emotional challenges. For example, the distress of potential infertility can heighten the emotional burden of sexual health changes, making it harder for individuals to navigate these already complex issues. Lastly, the financial burden of managing long-term sexual and reproductive health complications was mentioned as “*finances and costs are extreme…it’s a real barrier”*, as participants pay monthly fees for the storage of sperm, eggs, and/or embryos, pelvic floor therapy, sexual health counselling, and potential surrogacy fees.

### Thematic area II: external impacts of AYA cancer diagnosis and treatment

#### Theme 1: how dynamics in romantic and sexual relationships are changed (Experience)

Participants reported changes in the dynamics of their romantic and sexual relationships due to the impact of cancer and its treatment on their sexual health. From the participants’ perspective, a contributor to this dynamic change was that during treatment, they “*didn’t have sex at all*” particularly due to decreased libido but also because of weakness, fatigue, and focus on navigating treatment. Even after treatment, this decreased libido continued for some as participants *“felt like that part of [their] brain was off or gone dark”*, and having sex as “*out the window*”, “*obligation*”, or being “*like a play*,* you need to prepare for things…it’s not romantic and [they] don’t feel sexually attractive*”. With these changed dynamics came feelings of guilt as captured by this quote: “*I have had to navigate a lot him feeling really rejected. A lot of my concern was that if I were to say yes*,* how far do we have to go? Am I ready to go that far? Am I going to be able to manage it pain wise today*,* emotionally*,* digestively? It just felt easier to say “no” but then we got to a point where it was just he’s afraid to try*,* I don’t know how to try either and you almost feel like strangers in some ways.”* Participants also shared feelings of obligation to engage in sexual activity as “*it just wasn’t something that really came to [their] mind except for feeling bad that [they] weren’t doing it with [their] partner*” as their partners would “*be heartbroken*” otherwise. However, some participants shared ways for navigating these changed dynamics, such as rebuilding an emotional connection through going on a date at least once a week and integrating “*small gestures of affection throughout the day*,* like hugs and holding hands*”. Participants also shared the importance of communicating their needs as well as limitations to their partners.

For some participants, their partners taking on the role of a caregiver was another change to the relationship that was brought on by cancer and its treatment. As AYA cancer patients’ partners are often also young adults themselves, they have little lived experience on how to be a caregiver. This unexpected role added pressures on the relationship, which can create emotional distance, especially if the relationship is a new one, making the “*relationship go from traveling down the road at a gentle pace to running together…seeing if [they’re] going to make this or not*”, which is often the case with AYAs. A participant described the impacts of these changes in their marriage while juggling new parental responsibilities, a common scenario with AYAs: “*We had a 5-month old and we were brand new parents at the time and trying to figure that out. He admitted to me after the fact there was resentment on his side because he had to step into the role of being both parents. I was not physically feeling well…we were just in survival mode*” (Cohort 1, colorectal cancer at 35). Participants were conscious about their partners’ mental health, particularly feelings of anxiety and worry as they take on caregiver roles explaining that there is “*an extra burden that’s fallen on [them] because [they] feel like [they] need to check in*” and provide support, even though they’re “*not trained to provide that kind of support*”. In turn, participants, shared that they do “*not tell [them] things because [they] don’t want [the partner/caregiver] to freak out more*,* which then puts a big impact on the relationship*”. Participants’ self-consciousness also extended to their and their partners’ sexual health, in describing the embarrassment when their partner had to engage in sensitive caregiver activities like “*bowel movement tracking*” and felt like they “*didn’t want to have sex after doing this*”. Finally, participants described their partners as undergoing physical exhaustion as they must “*wait on [the patient] hand on feet… bust ass at work*,* get the groceries*,* do all the laundry*,* cook dinner and now you’re trying to find that spark*.” Participants worried that these impacts on their partners resulted in less time for their partners’ personal needs and interests, which may cause resentment and frustration. Some participants shared that “*stepping away from that caregiver role and more into the relationship role*” helped after treatment is complete, along with counselling to address “*the types of resentment that would come with cancer*”.

Finally, some participants reported challenges in navigating dating after cancer and its treatment, often due to the direct impacts on their sexual and reproductive health. For those who have experienced challenges with their ability to engage in sexual activity, they worry they would not be able find “*someone who is going to be okay with [their] body image*,* vaginal dryness*,* and pain with intercourse*” or reduced fertility. Participants also worried about how and when to disclose these cancer impacts with potential partners, as they felt it’s *“a lot of baggage to introduce to somebody”*. “*Are they going to be okay with the fact that I may not be able to have kids? Do I tell them right away*?” are questions that plagued AYA cancer patients. Fear of recurrence loomed large for patients as well, and it “*built into how [they] felt in relationships and if it felt fair to be in relationships*”. Some participants also described carrying feelings of betrayal and grief from a previous relationship affected by cancer into their new relationships as captured by this quote: “*I was in a long-term relationship leading up to my diagnosis that ended with me being cheated on because I was sick. It was very traumatic. I know it impacted a lot of my sexual desires*,* a lot of my overall body image and self-worth. Even now in dating and that sort of thing*,* it ends up me questioning if that’s going to happen again. There’s a level of trauma around being connected to a person. That definitely gets brought up when you’ve had a critical illness like cancer”* (Cohort 1, nasopharyngeal at 25 years old).

#### Theme 2: shifting family planning due to impaired sexual and reproductive abilities (Response)

Participants who have not yet started families and were no longer able to conceive or carry their own biological children expressed feelings of grief over having to change their family planning goals such as “*giving up the idea of having a baby naturally*”, “*saving money for IVF*”, or “*waiting* [*to see whether their fertility*] *might go back to normal”*. While there were levels of acceptance, participants felt that the forced decision to forego having biological children led to significant grief. A participant described being “*right at the point where [they] were kind of thinking about having kids*,* so the timing [of the cancer] was pretty unfortunate.*” We also observed the added strain on relationships resulting from changes in reproductive ability, with participants sharing fears about whether their partners would remain, asking questions such as: “*What if I can’t? What is he going to do? Is he going to stay with me?*”. When partners were supportive, participants had to make quick decisions regarding sperm banking, egg harvesting, and embryo freezing, describing the process as: “*You only have a few weeks to work it out. There’s not a whole lot more that you can do*,* so we just had to make a decision [regarding fertility preservation]*”. These were not without financial concern, as storage fees and in-vitro fertilization are *“$15*,*000*,* $20*,*000*,* $25*,*000 potentially with no guarantee of there being any kind of success at the end of that*”. While adoption is seemingly presented as an option to AYA cancer patients, some noted that having been diagnosed with cancer means they “*are not eligible for things like fostering and adopting until no evidence of disease for a minimum of 5 years*”, so this is less feasible than expected. In addition to these concerns, participants also found themselves feeling guilty about having children should they “*not be around for much longer*”, or if they have inherited a risk of cancer. Finally, the meaning of *“family*” changed, particularly for participants who have not yet had children. Some reflected on how being an aunt or uncle to children in their community can fill the need for family, and that “*you can still be a mom*,* it doesn’t have to be your biological child*”.

Participants who had already started families described “*coming to terms*” with not being able to grow their families more as reflected by this quote about having one child instead of the planned two or more, to “*enjoy and give [the one child] everything [they] can to enjoy life*”. Participants also worried about their ability to parent effectively given health challenges associated with cancer such as fatigue, feelings of depression, and anxiety, as described here: “*Can I be a mom*,* with all of the symptoms that I have? I have severe fatigue and I can’t multitask like I used to ‘cause of my chemo brain*,* brain fog and I’m not as healthy because of the weight gain*” (Cohort 8, breast cancer at 33). For those who were in the younger AYA age range, some had deprioritized building a family, as they felt that they had to “*play catch up for a few years*,” and their “*only goal right now is focusing on [themselves] and fostering [their] career.*”

#### Theme 3: how impaired sexual and reproductive health influence experiences of societal pressures (Experience)

For some participants, the impact of cancer and its treatment on sexual and reproductive health further amplified societal pressures, including that of maintaining normative sexual relationships as expressed by this quote: “*If I didn’t have cancer*,* I think we would be fine sexually. I don’t know how to repair it. I don’t know how to make it better. I’m trying but it’s tough*,” and “*feeling guilty and very alone*” when this was not realized. This guilt was exacerbated when participants explored masturbation, as they felt “*ashamed because [they] should be doing it with [their partner] and not re-exploring*”. This pressure was amplified as participants navigated sexual health resources, as described here: “*I found the very resources I had found that aren’t just cancer patient to cancer patient resources have been very much like*,* “Well you’re not going to be desirable if you can’t have this certain thing in your relationship.”…I feel like it really commodifies our bodies as something that is to be used by somebody else or to pleasure somebody else and doesn’t really take into account our own pleasure and if we feel good and if we’re having a good time.”*

Fertility and family planning are also associated with high societal pressure and expectations, particularly as these are major milestones for AYAs. Participants who chose to not have children, whether due to changing priorities or impaired fertility due to AYA cancer and its treatment, were met with scrutiny. One participant explained, “*I feel like the expectation from…my mom is that she wants to have grandchildren at some point. There’s an ongoing pressure point about what’s happening and*,* ‘What are the plans and what are you going to do about it?*’” Having a positive cancer prognosis and childbearing were also heavily intertwined for “*people”* (including family and friends) surrounding the patient as shared by a participant that “*hope and fertility got really wrapped up together where it was a win to have fertility there because it meant there was hope in some way*,* which made it loaded*”. Participants also shared that these “*people’s*” imposed expectations had “*a negative impact on [the patient’s] mental health*”. Participants who wanted to have children but had difficulty felt alienated from friends and social circles as “*a lot of friends were having kids and starting families*” while because of cancer, they had to “*accept that isn’t going to be the direction of [their] life and [they’re] not going to be able to relate…that’s not an option for [them]*”. Aside from fertility and family planning, another isolating factor for AYA cancer patients, particularly females, was premature menopause. Participants who experienced premature menopause induced by cancer treatment shared having many symptoms that their peers could not relate to, describing “*that at 31*,* none of my peers were talking about menopause at that time…they’re not talking about hot flashes or vaginal dryness or things like that. And it’s really hard to relate. You’re almost relating to an older cohort that’s like*,* you know*,* my mom’s age*,* that are dealing with those types of things*” (Cohort 6, ovarian cancer at 31 years old).

## Discussion

By using a novel serial focus group approach in a setting that fostered trust and community building, our study offers insights into the complex impacts of AYA cancer diagnosis and treatment on sexual and reproductive health. These impacts, which can be broadly described as both experiences and responses, affected patients inwards through direct effects on themselves and outwards in the context of their relationships and communities. These study findings have implications for validating AYA cancer patient experiences with highly sensitive and often stigmatized topics. Importantly, the findings have the potential to inform patient-oriented, intersectional approaches to address the complex impacts of AYA cancer on sexual and reproductive health.

Our study expands on the limited literature exploring sexual and reproductive health among patients with AYA cancer. In 2016, Frederick et al. explored sexual health through semi-structured interviews with 22 childhood and AYA cancer patients (10 women, 12 men) and showed that dysfunction resulted in interrupted psychosocial development, body image issues, and fertility concerns [16]. A 2021 qualitative study by Bentsen et al. conducted 12 interviews with women aged 20–35 and found that more support is needed for navigating fertility information. This study also highlighted that health care providers approach has a major impact on the patients consultation, and that inadequate and worrying information causes mistrust and frustration [28]. More recently in 2023, Bentsen et al. explored thoughts about fertility among 12 female AYA cancer patients and identified that patients had held onto hopes of having children, experienced time pressure, faced existential and ethical choices about family planning, and felt a loss of control over their bodies [29]. Aside from finding similar results, particularly with respect to impacts on sexual self-esteem, fertility, and sexual function, we addressed conceptual and methodological gaps of these prior studies by including male, cis-gender men, non-binary, and gender-diverse AYA perspectives. We also uncovered new perspectives, particularly with respect to grief and trauma, societal pressures and relationship impacts, and comparative experiences across demographic and clinical characteristics.

Also, an important finding in our study are participants’ perceptions of limited or lack of support for their sexual and reproductive health needs, which was reported in prior studies [30, 31]. It is important to contextualize these findings with those from prior research among providers. Specifically, in 2022 Albers et al. conducted interviews with six doctors and eight nurses working in AYA sexual health care and found that providers were unsure who is responsible initiating discussions on this topic, needed to find optimal timing for these conversations, sought more knowledge and training to enable discussions, and necessitated more informative material for AYAs [31]. Providing targeted education for health care providers that translate patient experiences as found in this study will inform comprehensive care. Further, we recommend that this education utilizes an intersectional lens that considers the identities of the providers and their patients, such as age, sex, gender, and cultural experience. This patient-oriented approach will increase accessibility, availability, and appropriateness of sexual and reproductive health resources and support for AYA cancer patients [32]. 

Lending strength to our study is the novel use of serial focus groups which helped build a sense of safety and community among participants; Indeed, the focus group format facilitated peer-to-peer information transfer, an integral component of AYA social networks. Additionally, our use of online methods increased accessibility, and targeted recruitment ensured a diverse range of participants across both demographic and clinical factors. Lastly, PRPs within the research team provided valuable insight at each stage of the research from study design (e.g., topic guide development and testing), data gathering (e.g., co-facilitating focus groups), to data interpretation. A potential limitation of the study is that the sensitivity of the topics may have caused selection bias as those who participated may have been more comfortable discussing sensitive topics in a group, while some AYAs might prefer to share their experiences privately. Additionally, the study population, which is predominantly White, educated, and in relationships, may not fully represent the diverse cultural and demographic experiences of all AYA cancer patients.

In summary, our study provides new insights into the complex and often overlooked impacts of AYA cancer on sexual and reproductive health. Specifically, we highlight the profound effects of cancer treatments on both physical and mental aspects of sexual and reproductive well-being, and how these challenges are compounded by societal expectations and relationship dynamics. These findings emphasize the need for targeted, patient-centered care that addresses the unique sexual and reproductive health concerns of AYA cancer patients.

Moving forward, it is crucial to develop and implement healthcare practices and policies that are responsive to these needs. Future research should focus on longitudinal studies that explore the long-term effects of cancer and its treatment on sexual and reproductive health, and how healthcare providers can best support AYA patients in navigating these challenges. Additionally, further exploration of the role of peer support, community-based interventions, and the integration of sexual health services into cancer care would be beneficial in shaping comprehensive, supportive care for AYA cancer patients.

## Supplementary Information

Supplementary Material 1.

## Data Availability

The data underlying this article will be shared on reasonable request to the corresponding author.

## References

[1] Scott AR, Stoltzfus KC, Tchelebi LT, et al. Trends in cancer incidence in US adolescents and young adults, 1973–2015. JAMA Netw Open. 2020;3(12):e2027738. 10.1001/jamanetworkopen.2020.27738. 33258907 10.1001/jamanetworkopen.2020.27738 PMC7709088

[2] Mercadante S, Vitrano V, Catania V. Sexual issues in early and late stage cancer: a review. Support Care Cancer. 2010;18(6):659–65. 10.1007/s00520-010-0814-0. 20237806 10.1007/s00520-010-0814-0

[3] Abbott-Anderson K, Kwekkeboom KL. A systematic review of sexual concerns reported by gynecological cancer survivors. Gynecol Oncol. 2012;124(3):477–89. 10.1016/j.ygyno.2011.11.030. 22134375 10.1016/j.ygyno.2011.11.030

[4] Gebauer J, Higham C, Langer T, Denzer C, Brabant G. Long-term endocrine and metabolic consequences of cancer treatment: a systematic review. Endocr Rev. 2019;40(3):711–67. 10.1210/er.2018-00092. 30476004 10.1210/er.2018-00092

[5] Geue K, Brahler E, Faller H, et al. Prevalence of mental disorders and psychosocial distress in German adolescent and young adult cancer patients (AYA). Psychooncology. 2018;27(7):1802–9. 10.1002/pon.4730. 29644783 10.1002/pon.4730

[6] Barnett M, McDonnell G, DeRosa A, et al. Psychosocial outcomes and interventions among cancer survivors diagnosed during adolescence and young adulthood (AYA): a systematic review. J Cancer Surviv. 2016;10(5):814–31. 10.1007/s11764-016-0527-6. 26920873 10.1007/s11764-016-0527-6 PMC5001943

[7] Zucchetti G, Bellini S, Bertolotti M, et al. Body image discomfort of adolescent and young adult hematologic Cancer survivors. J Adolesc Young Adult Oncol. 2017;6(2):377–80. 10.1089/jayao.2016.0067. 28112547 10.1089/jayao.2016.0067

[8] Yang Y, Li W, Wen Y, et al. Fear of cancer recurrence in adolescent and young adult cancer survivors: a systematic review of the literature. Psychooncology. 2019;28(4):675–86. 10.1002/pon.5013. 30703261 10.1002/pon.5013

[9] van Dorp W, Haupt R, Anderson RA, et al. Reproductive function and outcomes in female survivors of childhood, adolescent, and young adult cancer: a review. J Clin Oncol. 2018;36(21):2169–80. 10.1200/jco.2017.76.3441. 29874135 10.1200/JCO.2017.76.3441 PMC7098836

[10] Chao C, Bhatia S, Xu L, et al. Chronic comorbidities among survivors of adolescent and young adult cancer. J Clin Oncol. 2020;38(27):3161–74. 10.1200/jco.20.00722. 32673152 10.1200/JCO.20.00722 PMC7499612

[11] Sexual and Reproductive Health and Research (SRH). World Health Organization. https://www.who.int/teams/sexual-and-reproductive-health-and-research/key-areas-of-work/sexual-health/defining-sexual-health.

[12] Oveisi N, Cheng V, Brotto LA, et al. Sexual health outcomes among adolescent and young adult cancer patients: a systematic review and meta-analysis. JNCI Cancer Spectr. 2023;7(6). 10.1093/jncics/pkad087. 10.1093/jncics/pkad087 PMC10674049 37878813

[13] Oveisi N, Cheng V, Ellis U, et al. Reproductive Health Outcomes among Adolescent and Young Adult Cancer Patients: A Systematic Review and Meta-Analysis. Cancers (Basel). 2023;10(6). 10.3390/cancers15061707. 10.3390/cancers15061707 PMC10046594 36980593

[14] Benedict C, Shuk E, Ford JS. Fertility issues in adolescent and young adult cancer survivors. J Adolesc Young Adult Oncol. 2016;5(1):48–57. 10.1089/jayao.2015.0024. 26812452 10.1089/jayao.2015.0024 PMC4779291

[15] Keehnen JM, Vlooswijk C, Van Der Graaf WTA, Husson O, Dinkelman-Smit M. Fertility related concerns in male adolescent and young adult (AYA) cancer survivors from a multicenter cohort study (SURVAYA). Eur Urol. 2023;83:S316. 10.1016/S0302-2838(23)00275-0.

[16] Frederick NN, Recklitis CJ, Blackmon JE, Bober S. Sexual dysfunction in young adult survivors of childhood cancer. Pediatr Blood Cancer. 2016;63(9):1622–8. 10.1002/pbc.26041. 27163396 10.1002/pbc.26041

[17] Moore JB, Canzona MR, Puccinelli-Ortega N, et al. A qualitative assessment of body image in adolescents and young adults (AYAs) with cancer. Psychooncology. 2021;30(4):614–22. 10.1002/pon.5610. 33275802 10.1002/pon.5610 PMC8059971

[18] Kent EE, Smith AW, Keegan TH, et al. Talking about Cancer and meeting peer survivors: social information needs of adolescents and young adults diagnosed with Cancer. J Adolesc Young Adult Oncol. 2013;2(2):44–52. 10.1089/jayao.2012.0029. 23781400 10.1089/jayao.2012.0029 PMC3684139

[19] Matsui M, Taku K, Tsutsumi R, et al. The role of peer support in psychosocial outcomes among adolescent and young adult (AYA) cancer survivors. J Clin Oncol. 2020;38(15). 10.1200/JCO.2020.38.15_suppl.e22528.

[20] Yadav D. Criteria for Good Qualitative Research: A Comprehensive Review. Asia-Pacific Educ Researcher. 2021;(31). 10.1007/s40299-021-00619-0.

[21] Oveisi N, Cheng V, Taylor D, et al. Meaningful patient engagement in adolescent and young adult (AYA) Cancer research: A framework for qualitative studies. Curr Oncol. 2024;31(4):1689–700. 10.3390/curroncol31040128. 38668031 10.3390/curroncol31040128 PMC11049004

[22] Qualtrics Survey Software Version S. 2024. Qualtrics; 2024. https://www.qualtrics.com.

[23] Version 6. 0.11. Zoom Video Communications. Inc.; 2024.

[24] Saunders B, Sim J, Kingstone T, et al. Saturation in qualitative research: exploring its conceptualization and operationalization. Qual Quant. 2018;52(4):1893–907. 10.1007/s11135-017-0574-8. 29937585 10.1007/s11135-017-0574-8 PMC5993836

[25] International Q. NVivo. Version 14. 2024.

[26] Gale NK, Heath G, Cameron E, Rashid S, Redwood S. Using the framework method for the analysis of qualitative data in multi- disciplinary health research. BMC Med Res Methodol. 2013;13:117. 10.1186/1471-2288-13-117. 24047204 10.1186/1471-2288-13-117 PMC3848812

[27] Tannenbaum C, Greaves L, Graham ID. Why sex and gender matter in implementation research. BMC Med Res Methodol. 2016;(1): 145. 10.1186/s12874-016-0247-7. 27788671 10.1186/s12874-016-0247-7 PMC5084413

[28] Bentsen L, Pappot H, Hjerming M, Colmorn LB, Macklon KT, Hanghøj S. How do young women with cancer experience oncofertility counselling during cancer treatment?? A qualitative, single centre study at a Danish tertiary hospital. Cancers (Basel). 2021. 10.3390/cancers13061355. 10.3390/cancers13061355 PMC8002475 33802795

[29] Bentsen L, Pappot H, Hjerming M, Hanghøj S. Thoughts about fertility among female adolescents and young adults with cancer: a qualitative study. Support Care Cancer. 2023;31(7):421. 10.1007/s00520-023-07887-0. 37357225 10.1007/s00520-023-07887-0 PMC10290964

[30] Oveisi N, Khan Z, Brotto LA. A qualitative study of sexual health and function of females with pelvic cancer. Sex Med. 2023;11(2):qfac002. 10.1093/sexmed/qfac002. 36910701 10.1093/sexmed/qfac002 PMC9978583

[31] Albers LF, Bergsma FB, Mekelenkamp H, Pelger RCM, Manten-Horst E, Elzevier HW. Discussing sexual health with adolescent and young adults with cancer: a qualitative study among healthcare providers. J Cancer Educ. 2022;37(1):133–40. 10.1007/s13187-020-01796-0. 32557400 10.1007/s13187-020-01796-0 PMC8816785

[32] Elkefi S, Asan O. The impact of Patient-Centered care on Cancer patients’ QOC, Self-Efficacy, and trust towards doctors: analysis of a National survey. J Patient Experience. 2023;10:23743735231151533. 10.1177/23743735231151533. 10.1177/23743735231151533 PMC9869234 36698621

